# The Origin of the Plasma-Cell Heterogeneity

**DOI:** 10.3389/fimmu.2015.00005

**Published:** 2015-01-23

**Authors:** Catherine Pellat-Deceunynck, Thierry Defrance

**Affiliations:** ^1^INSERM, U892, Nantes, France; ^2^CNRS, UMR 6299, Nantes, France; ^3^Université de Nantes, Nantes, France; ^4^CHU, Nantes, France; ^5^Centre International de Recherche en Infectiologie, Lyon, France; ^6^INSERM U1111, Lyon, France; ^7^Ecole Normale Supérieure de Lyon, France; ^8^Université Lyon1, Lyon, France; ^9^CNRS, UMR5308, Lyon, France

**Keywords:** plasma cell, B-cell, cell cycle, IL21, autophagy, B1, autoimmunity, myeloma

Plasma cells (PCs) are terminally differentiated B-cells producing large amounts of immunoglobulins (Igs). In humans, most circulating Igs are produced by bone marrow PCs. PCs differentiate from naïve or memory B-cells usually activated by specific antigens. It is still controversial whether the regulation of PC numbers and the “active” *in vivo* Ig diversity depend or not on non-specific reactivation of B-cells during infections. Depending on the stimulus (T-independent/T-dependent antigen, cytokines, and partner cells) and B-cell types (naïve or memory, circulating or germinal center, lymph nodes or spleen, and B1 or B2), both the phenotype and isotype of PCs differ suggesting that PC diversity is either linked to B-cell diversity or to the type of stimulus or to both. Knowledge of the mechanisms supporting PC diversity has important consequences for the management of: (i) plasma-cell neoplasias such as Multiple Myeloma and Waldenström’s Macroglobulinemia, (ii) vaccine protection against pathogens, and (iii) auto-immune diseases.

In this E-book, Drs. Xu and Banchereau review current knowledge on the molecules involved in PC generation induced by either dendritic cells or macrophages in T-dependent or T-independent B-cell activation (1). They further show how dendritic cells or macrophages provide a second signal (membrane-bound or soluble factors) that is required for PC generation. As proposed by the authors, increasing the crosstalk between Ag presenting cells and B-cells may lead to enhanced vaccine-induced Ab responses. On the contrary, interruption of this crosstalk might represent new strategies to treat auto-immune diseases. As splenectomy is the standard of care of patients with auto-immune thrombocytopenia, Dr. Mahévas and collaborators took the opportunity to characterize splenic PCs (2). They show that in patients who did not receive rituximab, spleen PCs had a plasmablast signature. By contrast, spleen PCs from patients who received rituximab had a plasma-cell signature. These *in vivo* findings suggest that short-lived immature PCs differentiated into long-lived PCs *in situ*, raising the question of the existence of splenic niches for long-lived PCs. In the spleens of naïve mice, Dr. Holodick and collaborators assess the origin and function of the small CD138+ B1a cell population. They show that this population is likely responsible for a substantial portion of natural IgM that differs from IgM produced by other B1a cell subsets, underlying the heterogeneity of IgM-secreting cells within B1a cells (3). Dr. Cunningham and collaborators review current knowledge on B1b cells in humans and mice, and discuss how B1 and B2 cells might interplay during the antibody response to proteins like porins from pathogenic microbes, which induce both classical T-dependent and T-independent response (4).

Long-lived PCs are usually found within bone marrow niches, the composition of which is still a matter of debate. Although bone marrow niches are known to provide favorable environments for PC survival, Dr. Tooze explores how a self-renewal model could account for the maintenance of long-lived PCs within niches (5). Indeed, Dr. Tooze describes how regular re-entry into cell cycle could account for PC maintenance. Independently of possible self-renewal, long-life of PCs involves several mechanisms such as autophagy. Indeed, as professional secretory cells dedicated for massive synthesis, assembly, and secretion of Abs, PCs display high ER stress (6). Using mice knocked-out for atg5, Drs. Oliva and Cenci show that both generation and long-life of PCs was decreased in the absence of autophagy, highlighting the role of protein catabolism (6). Generation of long-lived PCs requires cytokines and growth factors. Dr. Moens and collaborators provide an overview of *in vitro* studies (mouse and human origin) that evaluated the role of the different cytokines in inducing differentiation of distinct B cell subsets into PCs. They underline the central role of IL21 in PC generation from naïve B-cells (7). IL21, as other cytokines and growth factors, modulates the expression of Bcl2 molecules, and in that way the survival/death threshold of PCs (7). In myeloma cells, the expression of the pro (BH3-only, Bax, Bak) and anti-apoptotic (Bcl2, Mcl1) molecules of the Bcl2 family is different according to the molecular classification of patients with multiple myeloma, as shown by Drs. Gomez-Bougie and Amiot, who conclude that the apoptosis threshold in myeloma differs greatly between subsets of patients (8). Myeloma cells also display a wide heterogeneity with regard to phenotype. Dr. Robillard and collaborators describe the diversity of myeloma immunophenotypes, which is useful for the evaluation of minimum residual disease after treatment and represents a source of targets for antibody-based therapy in subsets of patients (9).

The nine contributions in this topic describe the heterogeneity of PCs and discuss mechanisms involved in heterogeneity. Altogether, these contributions provide an overview on mechanisms involved in PC cell diversity in the context of normal and pathologic Ab responses.

## Conflict of Interest Statement

The authors declare that the research was conducted in the absence of any commercial or financial relationships that could be construed as a potential conflict of interest.
